# The dual crisis of coronary thrombosis and sepsis in JAK2-mutated essential thrombocytosis: A clinical case report

**DOI:** 10.1097/MD.0000000000046996

**Published:** 2026-01-16

**Authors:** Fake Liu, Ting Fang, Zongqian Wu

**Affiliations:** a Department of Critical Care Medicine, Jiang’an County People’s Hospital, Yibin, Sichuan, China; b Department of Radiology, Jiang’an County People’s Hospital, Yibin, Sichuan, China; c Department of Oncology, Zhongjiang County People’s Hospital, Zhongjiang, Sichuan, China.

**Keywords:** essential thrombocytosis, infection, myeloproliferative neoplasmas, myocardial infarction, sepsis

## Abstract

**Rationale:**

Essential thrombocytosis (ET) is a myeloproliferative neoplasms (MPNs) primarily caused by JAK2 gene mutations. The condition is characterized by thrombosis or hemorrhage as its main complications, with infection posing a significantly elevated risk of mortality. The dual crisis of acute myocardial infarction and sepsis occurring simultaneously is extremely rare but fatal. Identifying and properly managing these rare yet severe complications is of paramount importance. This case report aims to emphasize the importance of timely identification of primary thrombocytosis and correct intervention of acute myocardial infarction and sepsis complications, especially early coronary angiography stent placement, anticoagulation-hemorrhage and sepsis management, which can not only improve patient prognosis but also effectively reduce the risk of death.

**Diagnoses:**

A 79-year-old female patient presented with palpitations and abdominal pain. An electrocardiogram showed ST-segment elevation in the inferior and anterior walls, indicating a ST-segment elevation myocardial infarction (STEMI). The elevated white blood cell count (36.92 × 10^9^/L) and procalcitonin (PCT) level (6.26 ng/mL) indicated septicemia markers, while imaging studies revealed intestinal gas accumulation and ascites; sepsis was confirmed. The platelet count peaked at 1357 × 10^9^/L, and genetic testing for MPNs revealed an 80% mutation in JAK2 V617F, ET was confirmed.

**Interventions:**

Emergency coronary stent implantation was performed, along with piperacillin-tazobactam antibiotic therapy and controlled fluid resuscitation. Treatment included antiplatelet therapy, anticoagulation therapy, and hydroxyurea.

**Outcomes:**

The patient’s condition improved, with complete disappearance of symptoms of palpitations and abdominal pain, normal infection indicators, and smooth discharge. One month later, the platelet decreased to 439 × 10^9^/L, and no bleeding or thrombosis complications occurred.

**Lessons:**

This case suggests that JAK2 gene testing should be conducted for unexplained thrombocytosis; occult infection must be investigated when myeloproliferative neoplasm patients develop leukocytosis; maintaining therapeutic balance is crucial in the dual crisis of thrombosis and sepsis.

## 1. Introduction

The annual incidence of essential thrombocytosis (ET) is approximately 1.5/100,000, and it is characterized by excessive thrombopoiesis. Patients with ET faced an 11% higher risk of arterial thrombosis and a 7% increased risk of venous thrombosis. Furthermore, those with mutations in the JAK2 V617F gene exhibited a heightened risk of cardiovascular events.^[1]^ In patients with ET, ST-segment elevation myocardial infarction (STEMI) as the initial presentation is extremely rare, and the occurrence of concurrent occult sepsis is exceptionally uncommon.Cases of ET initially presenting with concurrent STEMI and occult sepsis are extremely uncommon, with only scattered reports of ET-related single complications (either STEMI or sepsis) in existing literature. This dual crisis presents significant challenges for treatment. This case indicates that we should remain vigilant for ET-induced fatal myocardial infarctions and sepsis, and balance the risks of thrombosis and hemorrhage. It also emphasizes the importance of early identification and management of latent sepsis, as well as the protection of organ function.

## 2. Case presentation

**Figure 1.**
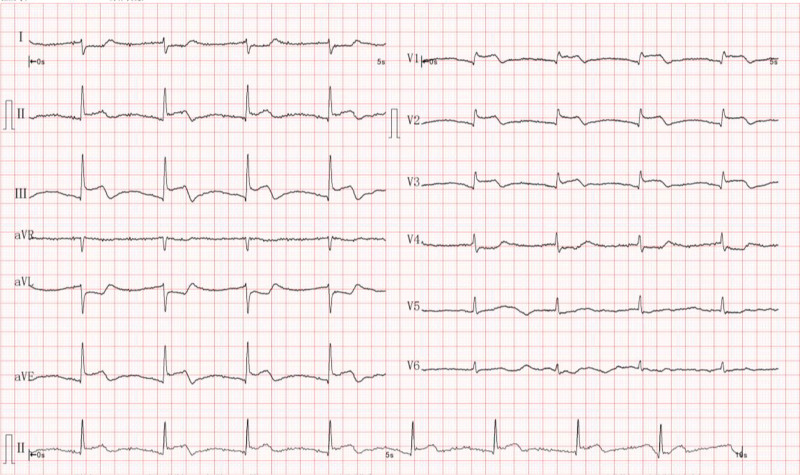
ST-segment elevation observed on her ECG in the inferior wall (II, III, aVF leads) and anterior septum (V1–V3 leads). ECG = electrocardiogram.

**Figure 2.**
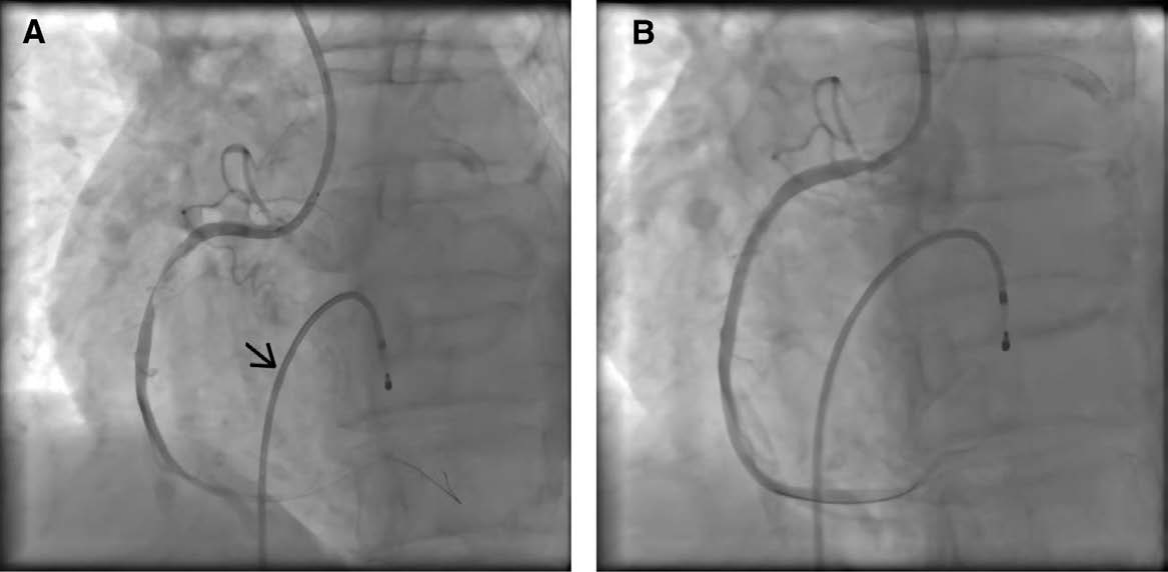
(A) → Temporary cardiac pacemaker lead, coronary angiography revealed a thrombus-like filling defect distal to the mid-segment of the right coronary artery. (B) Coronary stenting (3.0 29 mm) was performed.

A 79-year-old female patient was admitted to the emergency department with persistent palpitations (without chest pain) lasting 10 hours. Physical examination revealed blood pressure of 98/62 mm Hg, heart rate of 50 bpm, and respiratory rate of 21 bpm. No abnormalities were found during comprehensive systemic examinations. White blood cell count was 22.74 × 10^9^/L, while platelet count was significantly elevated to 784 × 10^9^/L. Standard electrocardiogram (ECG) showed ST-segment elevation in the inferior and anterior leads (Fig. 1). Although initial laboratory tests indicated normal troponin I levels, myocardial infarction should still be considered. Treatment included aspirin, clopidogrel, and heparin therapy. Two hours later, rechecked troponin I level was 4.75 ng/mL. Immediate coronary angiography revealed a thrombotic filling defect in the distal middle segment of the right coronary artery (Fig. 2A). The patient experienced an electrical storm during the procedure, and successful coronary angioplasty with stent implantation (3.0 × 29 mm) was performed after 4 electrical defibrillations (Fig. 2B). Transfer to intensive care unit (ICU) after surgery.

**Figure 3.**
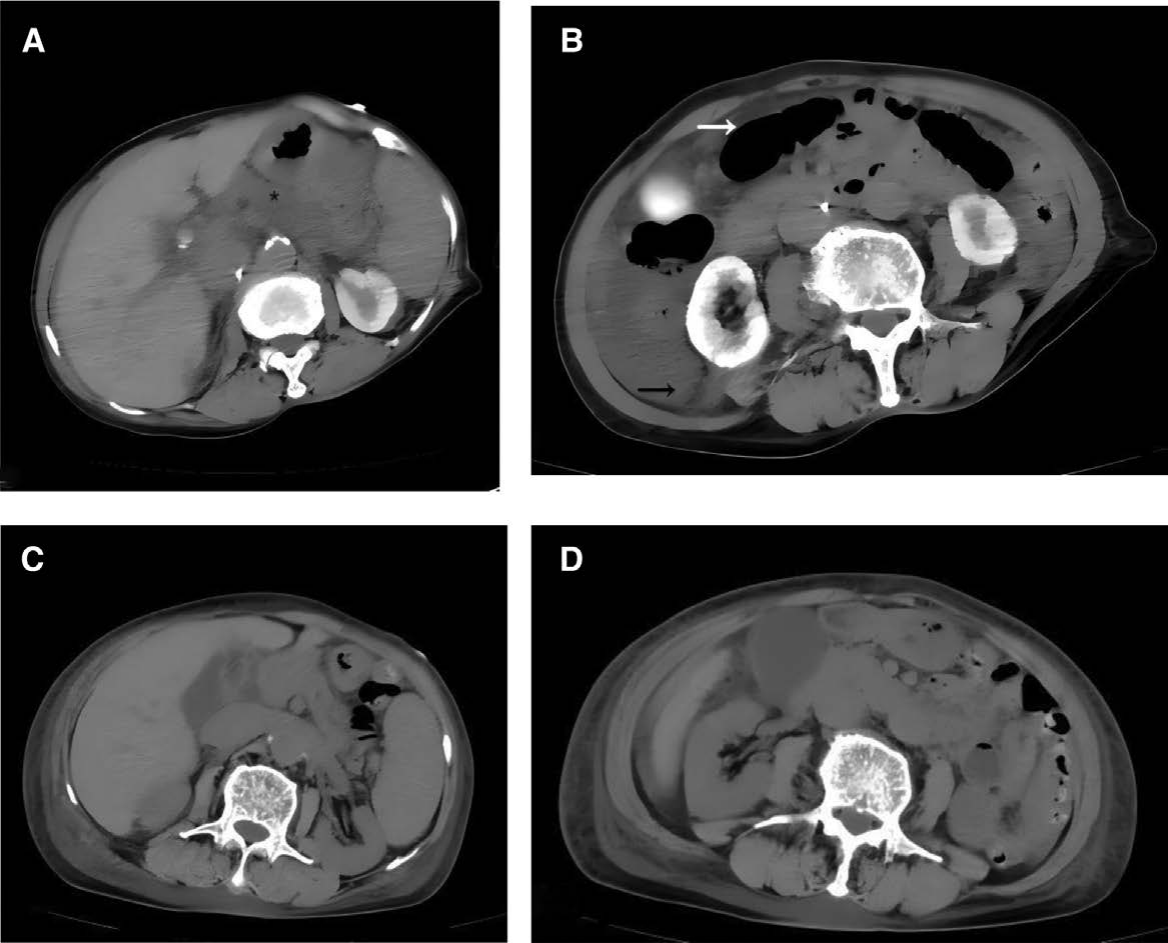
(A,B) Abdominal CT, * unclear outline of pancreatic body, → white arrow intestinal gas accumulation, black arrow ascites. (C,D) Abdominal CT, resolution of retroperitoneal effusions. CT = computed tomography.

Noradrenaline was administered at a rate of 0.8 µg/kg/min to maintain circulation. Blood gas analysis revealed a lactic acid level of 5.19 mmol/L. Bedside cardiac ultrasound indicated an ejection fraction of 60%. The temporary cardiac pacemaker functioned normally, and cardiogenic shock was ruled out. On the first day, the patient experienced abdominal pain. Laboratory tests revealed a white blood cell count of 36.92 × 10^9^/L, platelet count of 1357 × 10^9^/L, procalcitonin (PCT) level of 6.26 ng/mL, creatinine level of 204 µmol/L, and amylase level of 429.5 U/L. The abdominal computed tomography (CT) examination revealed a poorly defined outline of the pancreatic body, multiple retroperitoneal effusions, and intestinal gas accumulation (Fig. 3A and B). A gastrointestinal tract infection was suspected, and sepsis was present (the diagnosis of sepsis strictly adhered to the sepsis-3 criteria: SOFA score ≥ 2 (creatinine 204 µmol/L, norepinephrine > 0.1 µg/kg/min) and PCT > 2 ng/mL (6.26 ng/mL).^[2]^ Piperacillin tazobactam 4.5 g q12h were administered for anti-infection purposes, and fluid resuscitation was carried out under close monitoring to prevent shock and protect organ function (4000 mL on day 1, 3000 mL on day 2). Early anticoagulation therapy was initiated with aspirin 100 mg and clopidogrel 75 mg for antiplatelet effects, along with enoxaparin 1 mg/kg twice daily, to prevent the further formation of coronary artery thrombosis. 72 hours after admission (ICU day 3), The patient maintained stable circulatory status after discontinuing norepinephrine. Follow-up tests revealed a complete blood count of 19.18 × 10^9^/L, PCT level of 1.26 ng/mL, serum creatinine concentration of 90 µmol/L, and platelet count of 747 × 10^9^/L. A restrictive fluid replacement strategy (1500 mL daily) was implemented, along with enoxaparin at 1 mg/kg once daily, The patient was subsequently transferred from the ICU. One week later, follow-up tests showed a white blood cell count of 14.96 × 10^9^/L, PCT at 0.13 ng/mL, and platelet count at 874 × 10^9^/L. The abdominal CT scan results were normal (Fig. 3C and D). With infection under control but persistently elevated platelet count, the patient initiated ticagrelor antiplatelet therapy while discontinuing enoxaparin. Bone marrow biopsy and myeloproliferative neoplasm associated gene mutation testing were subsequently performed. Two weeks later, the results indicated mild myeloproliferative activity. Genetic analysis revealed an 80% JAK2 V617F mutation rate in the bone marrow, with negative BCR-ABL testing, confirming ET diagnosis. The treatment regimen was then adjusted to include 1 g hydroxyurea qd and continued ticagrelor 90 mg bid. One month after discharge, follow-up monitoring showed platelet count at 439 × 10^9^/L, with hydroxyurea dosage reduced to 0.5 g qd (Table 1).

**Table 1:** Key time points, key events and treatment measures.

Time node	Critical incident	Laboratory/Imaging results	Treatment measures
0 h after admission	Emergency visit, ECG: STEMI	Platelet 784 × 10^9^/L troponin I normal BP 98/62 mm Hg	Aspirin 300 mg, Clopidogrel 300 mg heparin
2 h after admission	TroponinI:4.75 ng/mL ECG: STEMI	Thrombus in the distal middle segment of the right coronary artery (Fig. 2A) intraoperative electrical storm	Implantation of a 3.0 × 29 mm stent and temporary pacemaker (Fig. 2B), followed by 4 defibrillation procedures
Admitted ICU	BP: 83/51 mm Hg	Blood gas analysis: PH 7.201 Lac 5.19 mmol/L EF 60%	Norepinephrine 0.8 μg/kg/min, multimodal monitoring
24 h after admission (ICU day 1)	Bellyache	White blood cell 36.92 × 10^9^/L procalcitonin 6.26 ng/mL creatinine 204 µmol/L platelet count of 1357 × 10^9^/L abdominal CT showed pancreatic blurring + retroperitoneal effusion (Fig. 3A and B)	Piperacillin tazobactam 4.5 g q12h monitored fluid resuscitation aspirin 100 mg + clopidogrel 75 mg enoxaparin 1 mg/kg q12h
72 h after admission (ICU day 3)	Cyclic stability	WBC 19.18 × 10^9^/L PCT 1.26 ng/mL creatinine 90 µmol/L, platelet count of 747 × 10^9^/L	Disable norepinephrine, restricted fluid (1500–1800 mL) enoxaparin 1 mg/kg qd transferred out of ICU
1 wk after admission	Infection control platelets remain high	WBC 14.96 × 10^9^/L PCT 0.13 ng/mL, platelet count of 874 × 10^9^/L Abdominal CT scan shows normal results (Fig. 3C and D)	Discontinue enoxaparin ticagrelor 90 mg bid, complete bone marrow smear bone marrow proliferative tumor-related gene mutation test
2 wk after admission	Remission	Platelet count of 680 × 10^9^/L 80% mutation in the JAK2 V617F gene of the bone marrow	Hydroxyurea 1 g qd ticagrelor 90 mg bid
Follow-up 1 mo	No complaints	Platelet count of 439 × 10^9^/L	Hydroxyurea 0.5 g qd

BP = blood pressure, CT = computed tomography, ECG = electrocardiogram, EF = ejection fraction, ICU = intensive care unit, Lac = lactic acid, PCT = procalcitonin, STEMI = ST-segment elevation myocardial infarction, WBC = white blood cell.

## 3. Discussion

This case report describes a rare dual crisis: a 79 year old female patient who had not previously been diagnosed with JAK2 V617F mutation-associated ET but simultaneously developed acute STEMI and sepsis. Current literature predominantly reports ET combined with single acute emergencies, such as ET with STEMI or ET with sepsis, without involving the triple pathological overlap of “thrombosis-infection-myeloproliferative disease.” This case focuses on the diagnosis and management of elderly ET patients with dual risks of myocardial infarction and sepsis, thrombo-hemorrhagic balance, and organ function protection, highlighting significant challenges in the diagnosis and treatment of myeloproliferative neoplasms (MPNs).

### 3.1. Thrombosis mechanism in ET

The arterial thrombosis associated with ET primarily stems from platelet hyperreactivity, leukocyte-platelet aggregation, and endothelial dysfunction-all driven by JAK2 gene mutations. The patient exhibited an unprecedented 80% allele frequency of JAK2 V617F, significantly exceeding the average 30 to 50% observed in typical polycythemia vera cases, which led to abnormal enhancement of thrombopoietin signaling and megakaryocyte proliferation. This molecular abnormality directly contributed to her development of extreme thrombocytosis (peak platelet count reaching 1357 × 10^9^/L), ultimately resulting in thrombosis in the distal middle segment of the right coronary artery (confirmed by coronary angiography, see Fig. 2A).^[3]^ Additionally, her persistent leukocytosis (initial 22.74 × 10^9^/L to peak 36.92 × 10^9^/L) indicated enhanced leukocyte-platelet aggregation, further exacerbating endothelial damage. This aligns with the characteristic ST-segment elevation observed on her ECG in the inferior wall (II, III, aVF leads) and anterior septum (V1–V3 leads) (see Fig. 1), which precisely corresponds to the classic clinical presentation of MPNs patients presenting with ST-segment elevation due to coronary microvascular thrombosis.^[4,5]^

### 3.2. Susceptibility to infection in MPNs

Infection-related mortality is often underestimated in patients with MPNs. Chronic inflammation, induced by clonal hematopoiesis, impairs neutrophil function and immune surveillance.^[6,7]^ The 80% JAK2 V617F allele burden likely drives this pro-inflammatory state, supported by laboratory parameters: PCT level of 6.26 ng/mL and white blood cell count reaching 36.92 × 10^9^/L. Imaging findings further support the infection origin: abdominal CT scans (Fig. 3A and B) revealed blurred pancreatic contour, multiple retroperitoneal effusions, and intestinal gas accumulation indicating intestinal barrier disruption and bacterial translocation secondary to microthrombus formation (complication of ET). Notably, the patient’s therapeutic response validated this mechanism: after one week of piperacillin-tazobactam 4.5 g q12h treatment, infection markers PCT decreased from 6.26 ng/mL to 0.13 ng/mL, retroperitoneal effusions/intestinal gas resolved (Fig. 3C and D), and renal function improved (creatinine from 204 µmol/L to 73 µmol/L). This demonstrates that the chronic inflammatory state of JAK2 mutant ET predisposes patients to occult sepsis.^[8]^

### 3.3. Therapeutic challenge

1. Thrombocytosis can arise from multiple etiologies. In this case, early diagnosis of myocardial infarction was confirmed, while the patient exhibited clinical manifestations of shock and significantly elevated infection markers. The thrombocytosis should be considered as potentially caused by hypovolemia and sepsis. Therefore, diagnosing ET poses significant challenges in early-stage cases of thrombocytosis without established medical history. Another critical diagnostic challenge in MPNs lies in distinguishing primary myeloproliferative “leukemia-like reactions” from infection-induced reactive leukocytosis. The patient’s elevated white blood cell count (36.92 × 10^9^/L) might be misattributed to idiopathic thrombocytosis alone. However, a PCT level of 6.26 ng/mL indicates an infection, whereas noninfectious MPNs typically show PCT levels < 0.5 ng/mL. Therefore, for complex cases such as idiopathic thrombocytosis, multidisciplinary and multidimensional comprehensive evaluation including clinical background plays a key role in guiding diagnosis and treatment.^[9]^
2. In this case, the JAK2 V617F allele burden (80% of cases) is a key driver of thrombotic risk. Sepsis further exacerbates the pathological conflict between thrombosis and hemorrhage. Sepsis activates neutrophil extracellular traps through pathogen-associated molecular patterns, providing a scaffold for von Willebrand factor and tissue factor, thereby promoting microvascular thrombosis.^[10]^ Concurrent with the thrombotic risk is the inherent bleeding tendency of ET.This presents substantial challenges in thrombo-hemorrhagic management when “ET + STEMI + Sepsis” coexist as a triple pathological burden. We implemented a dual scoring system for risk quantification: IPSET-thrombosis score: 79 years old (2 points) + JAK2 mutation (1 point) + recent STEMI (2 points), totaling 5 points indicating extremely high risk; HAS-BLED score: >75 years old (1 point) + renal insufficiency (1 point), totaling 2 points indicating moderate bleeding risk. This “high thrombosis + moderate bleeding” risk profile provides precise guidance for treatment decisions, prioritizing thrombus control while strictly preventing bleeding.^[11,12]^ For STEMI combined with hypercoagulability, initial dual antiplatelet therapy (aspirin + clopidogrel) was administered, Anticoagulant therapy was initiated with enoxaparin (1 mg/kg bid). As the condition stabilized, the dose was adjusted to enoxaparin (1 mg/kg qd) on day 2, followed by discontinuation after one week. The diagnosis of ET was confirmed through bone marrow biopsy and myeloproliferative nevus-related gene mutation testing. After achieving control of sepsis (PCT < 0.5 ng/mL), hydroxyurea (1 g daily) was administered to effectively avoid the risk of acute-phase immunosuppression. This treatment protocol was developed based on the patient’s elevated thrombotic risk (IPSET score 5) and persistent thrombocytosis, aligning with the European Thrombocytosis Society guidelines recommending “proactive cellular ablation for high-risk patients.” Ultimately, this strategy successfully reduced platelet targets below 600 × 10^9^/L without any bleeding events.^[13]^
3. Fluid resuscitation is an integral component of sepsis treatment; however, aggressive fluid replacement can lead to volume overload following STEMI. Therefore, carefultitration, guided by multi-index monitoring methods such as lactate clearance rate, is essential.^[14]^

## 4. Conclusions

The dual complication of myocardial infarction and sepsis in patients with ET is extremely rare, and this case has the following implications: Unexplained thrombocytosis (platelet count > 450 × 10^9^/L), even in the absence of a history of MPNs, requires JAK2 gene testing according to ICC guidelines; Thrombosis and sepsis crises can occur simultaneously in patients with ET. For MPNs presenting with leukocytosis, it is crucial to guard against occult infections; PCT detection and imaging are key methods for differentiation.

## Author contributions

**Methodology:** Zongqian Wu.

**Resources:** Ting Fang.

**Supervision:** Zongqian Wu.

**Writing – original draft:** Fake Liu.

**Writing – review & editing:** Fake Liu.
